# Dental Depot

**Published:** 1861-11

**Authors:** 


### DENTAL DEPOT.

We take pleasure in referring to Dr. J. M. Brown's Dental Depot, as one in which a fine stock of dental goods may at all times be found. This is the oldest Dental Depot in the West, and from the Doctor's long and ample experience in the purchase and sale of dental goods, he can come as near anticipating the wants of the dentist as any man we wot of. The Doctor's recent illness somewhat interrupted his close personal attention to the store, but his assistant, Mr. H., a man of undoubted ability and efficiency in that line, is always on hand. We are pleased to know that the Doctor is now able, and will henceforth give his closest attention to supplying the wants of his patrons. T.

